# TYK2 Kinase Activity Is Required for Functional Type I Interferon Responses *In Vivo*

**DOI:** 10.1371/journal.pone.0039141

**Published:** 2012-06-18

**Authors:** Michaela Prchal-Murphy, Christian Semper, Caroline Lassnig, Barbara Wallner, Christian Gausterer, Ingeborg Teppner-Klymiuk, Julianna Kobolak, Simone Müller, Thomas Kolbe, Marina Karaghiosoff, Andras Dinnyés, Thomas Rülicke, Nicole R. Leitner, Birgit Strobl, Mathias Müller

**Affiliations:** 1 Institute of Animal Breeding and Genetics, University of Veterinary Medicine, Vienna, Austria; 2 Biomodels Austria, University of Veterinary Medicine, Vienna, Austria; 3 Genetic Reprogramming Group Agricultural Biotechnology Center, Gödöllö, Hungary; 4 Department for Agrobiotechnology IFA Tulln, University of Natural Resources and Life Sciences, Vienna, Austria; 5 Institute of Laboratory Animal Science, University of Veterinary Medicine, Vienna, Austria; 6 Molecular Animal Biotechnology Laboratory, Szent Istvan University, Gödöllö, Hungary; 7 BioTalentum Ltd., Gödöllö, Hungary; National Jewish Health and University of Colorado School of Medicine, United States of America

## Abstract

Tyrosine kinase 2 (TYK2) is a member of the Janus kinase (JAK) family and is involved in cytokine signalling. *In vitro* analyses suggest that TYK2 also has kinase-independent, i.e., non-canonical, functions. We have generated gene-targeted mice harbouring a mutation in the ATP-binding pocket of the kinase domain. The *Tyk2* kinase-inactive (*Tyk2^K923E^*) mice are viable and show no gross abnormalities. We show that kinase-active TYK2 is required for full-fledged type I interferon- (IFN) induced activation of the transcription factors STAT1-4 and for the *in vivo* antiviral defence against viruses primarily controlled through type I IFN actions. In addition, TYK2 kinase activity was found to be required for the protein’s stability. An inhibitory function was only observed upon over-expression of TYK2^K923E^ *in vitro. Tyk2^K923E^* mice represent the first model for studying the kinase-independent function of a JAK *in vivo* and for assessing the consequences of side effects of JAK inhibitors.

## Introduction

Tyrosine kinase 2 (TYK2) belongs to the Janus kinase (JAK) family of non-receptor tyrosine kinases that, in mammals, additionally comprises JAK1-3 [1], [2]. JAKs associate with a variety of cytokine and growth factor receptors and upon ligand binding undergo auto- and/or cross-phosphorylation. Activated JAKs phosphorylate receptor chains and members of the signal transducer and activator of transcription (STAT) family. Phosphorylated STATs are homo- or heterodimers and translocate to the nucleus to initiate transcription. This is referred to as the linear – i.e. canonical – JAK-STAT signalling pathway [3]. Functionally, TYK2 was first identified as crucially contributing to type I interferon (IFNα/β) responses [4]. Murine and human cells deficient for TYK2 were instrumental in defining additional biological functions of TYK2 in signalling for a selection of cytokines [5]. Three groups have used gene targeting to create mouse models for *Tyk2* deficiency [6], [7], [8] and an additional model is provided by the naturally occurring *Tyk2* mutant strain B10.Q-H^2q^/Sgj (B10.Q/J) [9]. A human fibrosarcoma cell line lacking TYK2 was used in the majority of early studies on the protein’s functions [4], [10]. Recently, a patient with *TYK2* deficiency has been reported and initial studies confirm most findings from mutant mice and human cell lines, although they also pinpoint some differences between species [11].

Type I IFNs comprise several IFNα subtypes and one IFNβ and signal through IFNAR1 associated with TYK2 and IFNAR2/JAK1. IFNAR engagement primarily activates STAT1/2 heterodimers, which activate transcription together with IFN regulatory factor (IRF) 9. Cell type-specific type I IFN responses are mediated through additional activation of STAT3-6 [12], [13]. In addition to this canonical JAK-STAT pathway, alternative transcription factors are activated and there is cross-talk with other pathways – i.e. non-canonical signalling [14], [15]. *TYK2* deficiency in the human fibrosarcoma cell line [4] and in T cells of a patient carrying a homozygous mutation of the *TYK2* gene [11] leads to unresponsiveness to IFNα. By comparison, *Tyk2*-deficient mice have a reduced IFNα/β response. This has been attributed to a strong reduction of IFNAR1 surface levels in human *TYK2*-deficient cells, while mutant murine cells express unchanged IFNAR1 levels [5].

TYK2 shares with the other JAKs the conserved structure of seven JAK homology (JH) domains, wherein the C-terminal JH1 and JH2 encode the kinase and pseudokinase domain, respectively, and the N-terminal parts provide protein-protein interaction domains [1], [16]. The JH1 domain shows all the characteristics of a classical tyrosine kinase [17], [18], including conserved activation loop tyrosines and the ATP-binding residues. The mutation of either of these residues results in an impairment of catalytic activity [19], [20]. JH2 exerts regulatory functions with specific point mutations either abolishing or increasing the catalytic activity of TYK2 [21], [22], [23], [24]. JAKs may also actuate biological functions independently of their catalytic activity. To date, the best described effects relate to the *in vitro* stabilization of receptors and seem to be restricted to distinct receptor/JAK combinations. TYK2 stabilizes human IFNAR1 independently of its kinase domain [25], [26], and similar functions are described for other JAKs [27], [28]. In addition, kinase-independent functions of JAKs have been reported in the context of signal pathway crosstalk and mitochondrial functions [29], [30], [31]. Hence, the description of the full spectrum of JAK activities requires a consideration not only of kinase-dependent functions but also of non-canonical functions.

To dissect the canonical and non-canonical functions of TYK2 *in vivo* we gene-targeted the *Tyk2* locus, introducing a point mutation into the exon encoding the ATP-binding pocket. The resulting *Tyk2* kinase-inactive (*Tyk2^K923E^*) mice appear phenotypically normal in comparison to wild-type (WT) littermates. Analysis of the IFNα/β responses *in vitro* and *in vivo* revealed that (i) TYK2 kinase activity is essential for unperturbed signalling and (ii) the kinase-inactive protein exerts no inhibitory effects. Unexpectedly, we found a dependence of TYK2 protein stability on the JH1-mediated kinase activity. This might be of particular interest when considering the use of pharmacological TYK2 inhibitors in future clinical settings.

## Results

### Generation of *Tyk2* Kinase-inactive Mice

**Figure 1:**
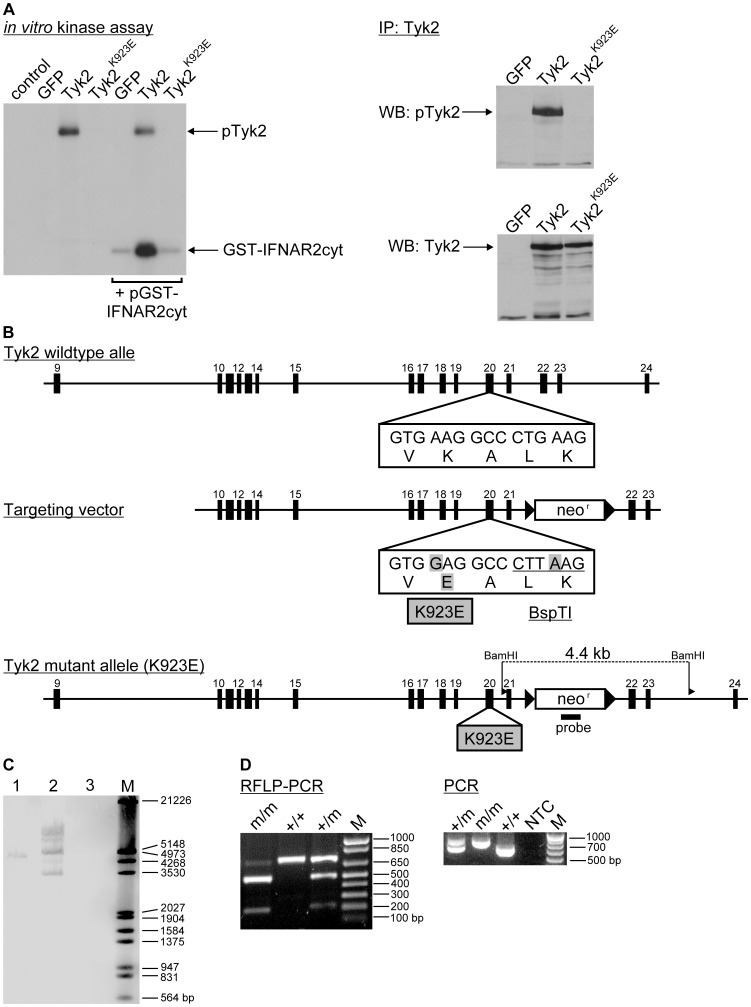
TYK2^K923E^ is enzymatically inactive and generation of *Tyk2^K923E^* mice. A. The *in vitro* kinase activity assay was performed in a TYK2-deficient cell line transiently transfected with plasmids encoding GFP, wild-type TYK2 or kinase-inactive TYK2^K923E^. TYK2 and TYK2^K923E^ proteins were immunoprecipitated from cell extracts and subjected to an *in vitro* kinase assay using GST-IFNAR*cyt* as an exogenous substrate (left panel). TYK2 was immunoprecipitated from whole cell extracts and Western Blot analysis performed to detect phosphorylated TYK2 (pTyk2, upper right panel) or TYK2 protein (lower right panel). B. Scheme of the murine *Tyk2* locus from exons 9-24 (black boxes). The point mutations introduced in exon 20 resulting in the amino acid exchange K>E and the introduction of the BspTI restriction endonuclease site are depicted. The neomycin resistance cassette (*neo^r^*, white box) flanked by loxP sites (black triangles) was inserted into the intron sequence between exons 21 and 22. The lower scheme shows the targeted locus with the restriction sites important for Southern blot analysis. Note that after germline transmission the *neo^r^* cassette was excised to leave a single loxP site in the mutated allele. C. Southern blot analysis using a non-radioactively labelled 471 bp *neo^r^* probe verified correct targeting and lack of heterologous integration in the ES cell clone 1, whereas two other clones (2 and 3) were not correctly targeted. D. DNA from WT (+/+), heterozygous (+/m) or homozygous *Tyk2^K923E^* (m/m) mouse tails was used to amplify a 710 bp fragment with primers surrounding exon 20. The amplicons were digested with BspTI resulting in a 498 bp and a 212 bp fragment only in the *Tyk2^K923E^* alleles. E. Conventional genotyping of mouse tails results in a 678 bp fragment corresponding to the WT and a 778 bp fragment specific for *Tyk2^K923E^*.

A kinase-inactive murine TYK2 analogous to the kinase-inactive human TYK2 protein [19] was generated by exchanging the conserved lysine (K923, NCBI GenBank: AF173032.1) in the kinase domain, which is essential for the catalytic activity, to glutamic acid (E) (Fig. 1B). The murine TYK2^K923E^ showed no enzymatic activity in an *in vitro* kinase assay (Fig. 1A), confirming data from human [19], [20] and murine [29] TYK2.

The gene-targeting vector for the generation of kinase-inactive *Tyk2* mice is depicted in Fig. 1B. Targeted ES cells were generated as described [32] and successful targeting of the *Tyk2* locus was verified by Southern Blot and PCR (Figs. 1C and D). Finally, the point mutations and vector integration sites were verified by DNA sequencing (data not shown). Six germline competent chimeras were obtained and gene-targeted line #29 was bred to *Tg(CMV-Cre)* mice [33] to remove the neomycin resistance cassette. Intercrossing of F1 generation mice demonstrated that B6N;129P2-*Tyk2^tm3(K923E)Biat^* (*Tyk2^K923E^*) mice were born at a normal Mendelian ratio, showed no apparent abnormalities and were fertile. The *Tyk2^K923E^* line was backcrossed to C57BL/6N background by speed congenics [34].

### The Stability of the TYK2 Protein Partially Depends on its Tyrosine Kinase Activity

**Figure 2:**
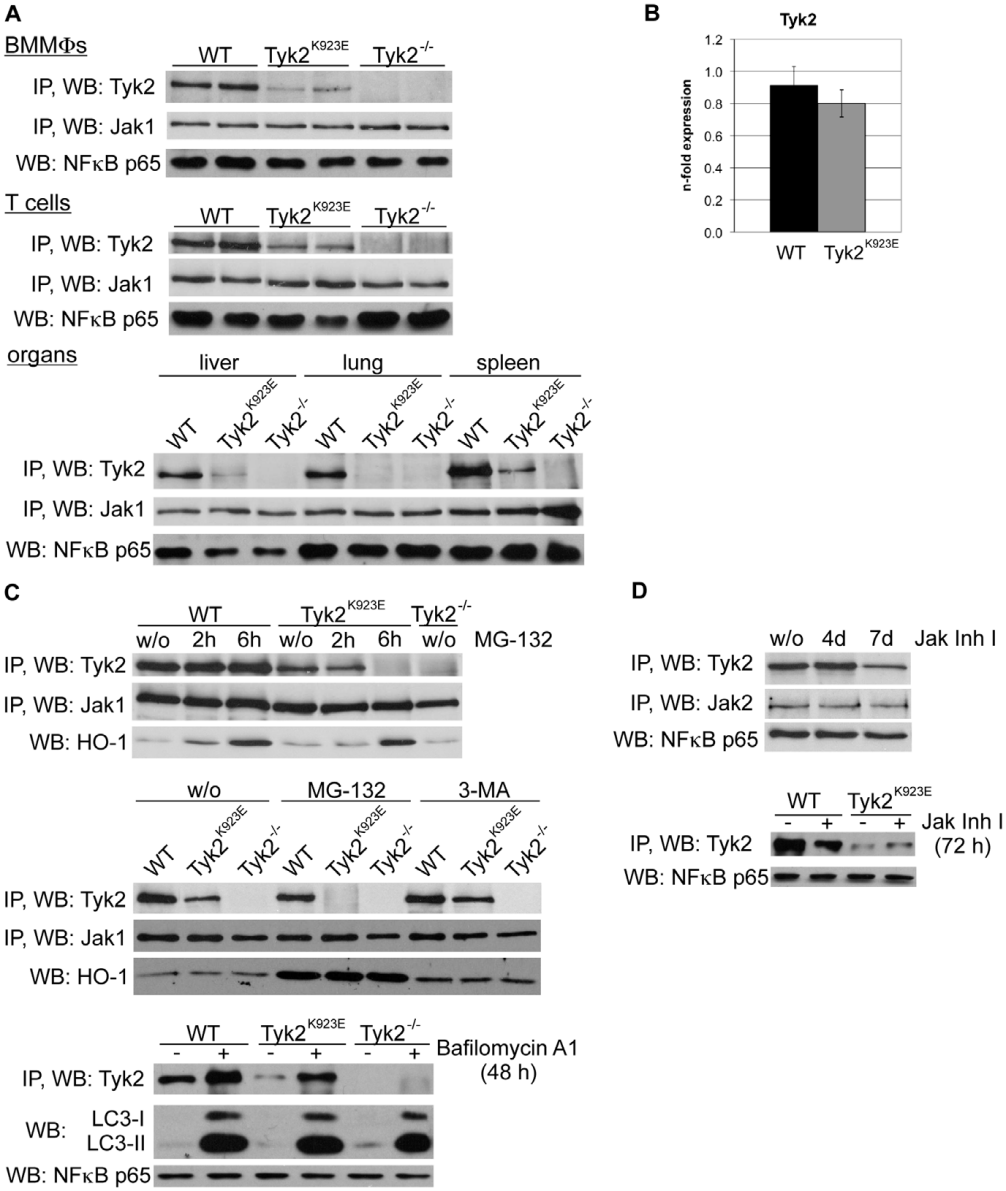
TYK2^K923E^ protein level is reduced and TYK2 differs organ-specifically. A. WT, *Tyk2^−/−^* and *Tyk2^K923E^* mice were used to prepare whole cell extracts from BMMΦs, T cells and various organs (as indicated). Levels of expression of TYK2 and JAK1 were determined by immunoprecipitation and Western blot analysis. NFκB-p65 was used as input control. TYK2^K923E^ protein levels were quantified using ImageJ software for Mac OS X (open source, http://rsb.info.nih.gov/ij/index.html) and were between 13% and 30% in BMMΦs and approximately 58% in T cells compared to WT. B. Total RNA was isolated from WT and *Tyk2^K923E^* BMMΦs and cDNA was used to analyse Tyk2 mRNA expression normalized to the housekeeping gene *Ube2D2*. Results from 4 independent experiments are shown (n = 6 per genotype). C. BMMΦs were treated with the proteasomal inhibitor MG-132 (50 µM), the autophagy-lysosome inhibitor 3-MA (10 mM) or the lysosome-acidification inhibitor bafilomycin A_1_ (80 nM) for the indicated period of time (upper panel), for 11 h (middle panel) or 48 h (lower panel). Whole cell extracts were used to determine TYK2 and JAK1 expression levels by immunoprecipitation and Western blot analysis. As a control, a Western blot for HO-1 was performed. D. From day 5 after isolation of WT BMMΦs, cells were treated with JAK inhibitor I (panJAK inhibitor; 15 nM upper panel and 300 nM lower panel) for the indicated period of time. TYK2 and JAK2 expression levels were analysed as described in (A and C); NFκB-p65 was used as input control.

Immunoprecipitation followed by Western Blot was performed with lysates from WT and *Tyk^K923E^* whole cells and organs to analyse TYK2 levels. A clear reduction of TYK2^K923E^ compared to WT protein levels was detected in all primary cells (bone marrow macrophages (BMMΦs) and T cells, Fig. 2A upper and middle panel; and murine embryonic fibroblasts (MEFs), data not shown) and organ extracts (liver, lung and spleen, Fig. 2A lower panel) we examined. Although immunoprecipitation and Western Blot technology are only semi-quantitative, it is noticeable that the WT TYK2 levels vary between organs, decreasing from spleen to lung and liver (Fig. 2A, lower panel), while JAK1 is more evenly expressed.

To eliminate the possibility that TYK2^K923E^ activity directly or indirectly influences its own expression and to verify the transcriptional integrity of the targeted locus, we analysed *Tyk2* mRNA by RT-qPCR. No significant differences in mRNA expression in BMMΦs isolated from WT or *Tyk2^K923E^* mice were detected (Fig. 2B). Similar results were obtained by analysing T cells and extracts from spleen, liver and lung (data not shown).

We next monitored the degradation of the mutated TYK2 protein. The ubiquitin-proteasome and the autophagy-lysosome systems are two major pathways triggering the degradation of proteins in mammalian cells [35], [36]. The pathways can be inhibited by treating cells with MG-132 or 3-methyladenine (3-MA), respectively. We tested the stability of TYK2^K923E^ in BMMΦs in the presence of MG-132. As a positive control we used heme oxigenase-1 (HO-1), which is stabilized when the proteasome pathway is blocked [37]. WT TYK2 levels remained unperturbed upon MG-132 treatment, while TYK2^K923E^ protein decreased after 2 h and was no longer detectable after 6 h of proteasome inhibition (Fig. 2C upper panel). In contrast, treatment of BMMΦs with 3-MA for 11 h nearly restored TYK2^K923E^ levels to the level of WT TYK2 (Fig. 2C middle panel). The enhanced TYK2^K923E^ degradation upon proteosomal inhibition can be explained by enhanced targeting to the autophagy-lysosomal machinery, as has been reported for other proteins [38]. In addition, we used bafilomycin A_1_ to block lysosomal acidification [39]. Again, treatment substantially increased TYK2^K923E^ protein level (Fig. 2C, lower panel). As positive control LC3-I and -II (microtubule-associated protein 1 light chain 3) accumulation was used [40]. To test whether the decreased TYK2^K923E^ stability is due to inactivation of the kinase rather than being a consequence of the point mutation that had been introduced, we treated WT cells with a panJAK inhibitor at different concentrations and time periods (Fig. 2D, upper and lower panel). A clear reduction of TYK2 protein was observed, although JAK2 levels remained stable (Fig. 2D). In conjunction, our results suggest that TYK2’s kinase activity is required to prevent its lysosomal-mediated degradation.

### *Tyk2^K923E^* and *Tyk2^−/−^* Innate and Adaptive Immune Cells Show Similar Impairment of IFNβ-induced STAT Activation

**Figure 3:**
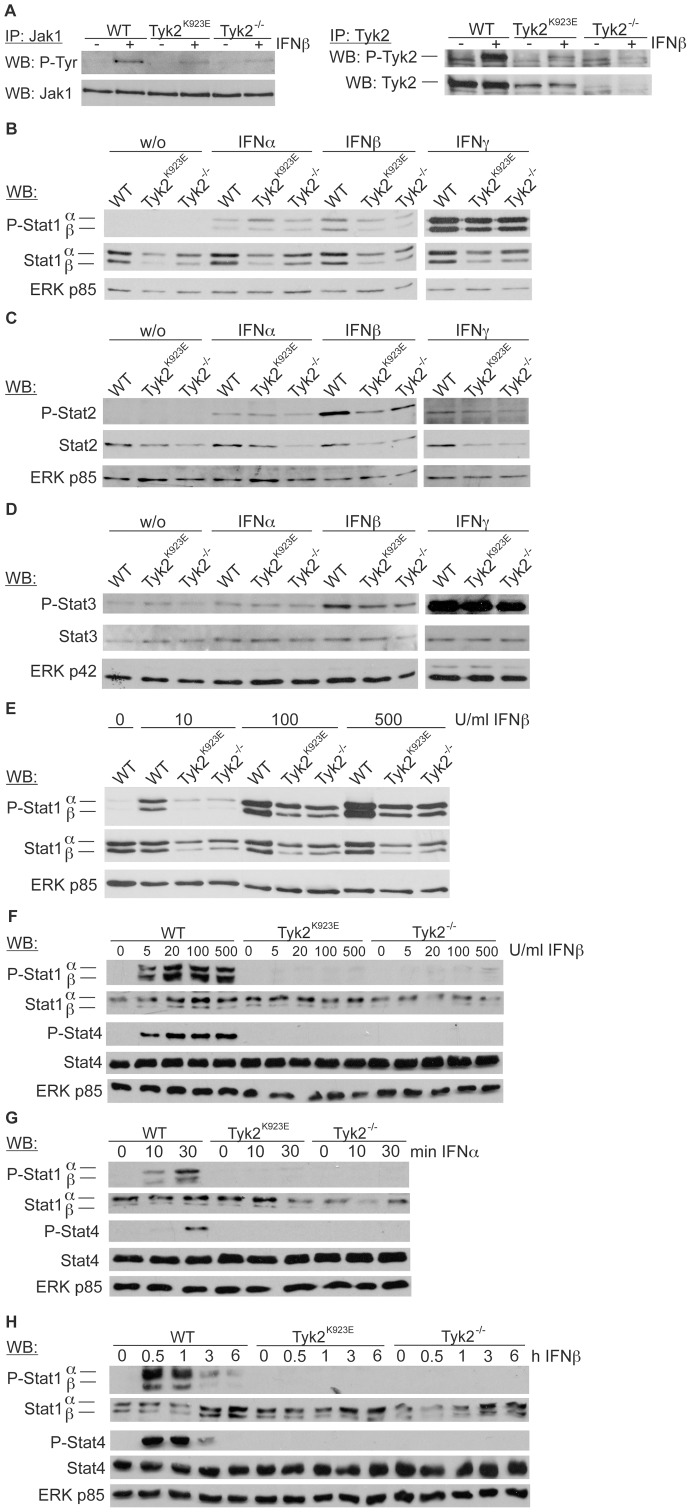
IFN treatment leads to similar activation of JAKs and STATs in *Tyk2^K923E^* and *Tyk2*-deficient cells. BMMΦs were treated with IFNβ (500 U/ml) for 20 min or left untreated. Whole cell extracts were used to determine levels of JAK1 tyrosine phosphorylation and JAK1 expression (left panel) and of TYK2 tyrosine phosphorylation and TYK2 expression (right panel) by immunoprecipitation and Western blot analysis. B.-D. BMMΦs were treated with IFNα (500 U/ml), IFNβ (100 U/ml) or IFNγ (100 U/ml) for 20 min or left untreated. Whole cell extracts were used to determine STAT1α/β tyrosine phosphorylation and levels of STAT1α/β expression (B), levels of STAT2 tyrosine phosphorylation and STAT2 expression (C) and levels of STAT3 tyrosine phosphorylation and STAT3 expression (D) by Western blot analysis. E. BMMΦs were treated with IFNβ (10, 100 or 500 U/ml) for 20 min or left untreated and Western blot analysis performed as described in (B). F. NK cells were treated with the indicated doses of IFNβ for 20 min or left untreated. Levels of tyrosine phosphorylation and protein expression of STAT1α/β and STAT4 were analysed by Western blot. G. NK cells were treated with IFNα (500 U/ml) for the times indicated and STAT1 and 4 analysed as described in (F); H. NK cells were treated with IFNβ (100 U/ml) for the times indicated and STAT1 and 4 analysed as described in (F); ERK p85 (B, C, E-H) and ERK p42 (D) served as a loading control.

Full activity of cytokine receptors binding TYK2 always depends on the binding of at least one additional JAK, so binding of TYK2 alone is insufficient to transduce signals. For example, JAK1 is the decisive upstream transphosphorylating kinase for TYK2 at the IFNAR or the gp130-utilising receptors [19], [41]. We have previously shown that JAK1 is phosphorylated on tyrosine residues upon treatment with IFNβ in the absence of TYK2, although the level of phosphorylation is reduced compared to WT [6]. We now assessed the phosphorylation state of JAK1 and TYK2 associated with IFNAR in IFNβ-treated WT and gene-targeted BMMΦs. JAK1 activation was detectable in the presence of kinase-inactive TYK2 and in the absence of TYK2 (Fig. 3A, left panel). Consistent with its reported function as a subordinate kinase, TYK2^K923E^ shows IFNβ-induced tyrosine phosphorylation (Fig. 3A, right panel). In addition to confirming the JAK1/TYK2 kinase hierarchy at the IFNAR complex, these findings suggest that the receptor architecture is intact in cells expressing kinase-inactive TYK2.

TYK2 deficiency leads to a partial impairment of STAT activation upon type I IFN stimulus in BMMΦs [6]. The ability of BMMΦs collected from *Tyk2*-deficient and kinase-inactive mice to activate STAT1-3 in response to IFNs was compared. In agreement with previous findings [6], [42] the levels of STAT1 and 2 proteins were decreased in *Tyk2^−/−^* BMMΦs; we found similar results in *Tyk2^K923E^* cells (Figs. 3B and C). In contrast, the levels of STAT3 protein were unaffected in *Tyk2*-mutant cells (Fig. 3D). Treatment of BMMΦs with IFNβ caused reduced tyrosine phosphorylation of the STAT1 isoforms, STAT2 and STAT3 (Figs. 3B–D); the extent of reduction was similar in *Tyk2^−/−^* and *Tyk2^K923E^* cells. The level of IFNα-induced STAT1-3 phosphorylation was just above detection limit and no gross differences between genotypes were observable. Treatment with IFNγ resulted in a slight reduction between WT and the two mutant genotypes in levels of activated STAT1 but not STAT3 (Figs. 3B and D). To investigate potential dose-dependent effects, we treated BMMΦs with varying amounts of IFNβ. There was a clearly dose-dependent increase of tyrosine-phosphorylated STAT1 in WT and *Tyk2*-mutated cells, although the mutant BMMΦs do not reach the levels of STAT1 activation exhibited by WT cells at least within the dose range tested (Fig. 3E).

In addition to STAT1 and 2, NK cells and T cells directly activate STAT4 upon IFNα/β treatment [13]. Analysis of IFNβ-treated NK cells revealed that lack of TYK2 and expression of mutant TYK2 equally impaired the phosphorylation of STAT1 and STAT4 (Figs. 3F and H). This was observed for doses up to 500 U/ml (Fig. 3F) and during the time course tested (Fig. 3H). Similar results were obtained with IFNα (Fig. 3G) and with ConA-activated splenocytes and T cells stimulated with IFNβ (data not shown). Note that levels of STAT4 do not differ between genotypes.

This experiment proves that upon IFNAR engagement (i) JAK1 is autophosphorylated in *Tyk2^−/−^* as well as in *Tyk2^K923E^* cells and (ii) JAK1 transphosphorylates TYK2^K923E^. Kinase-inactive TYK2 cannot compensate for the loss of TYK2 protein in the activation of STAT1-4 by type I IFN in innate and adaptive immune cells, while – at least at the levels detected – kinase-inactive TYK2 expressed *ex vivo* does not block JAK1 activity.

### IFN-induced Transcriptional Activation of Target Genes does not Differ between *Tyk2^−/−^* and *Tyk2^K923E^* Cells

**Figure 4:**
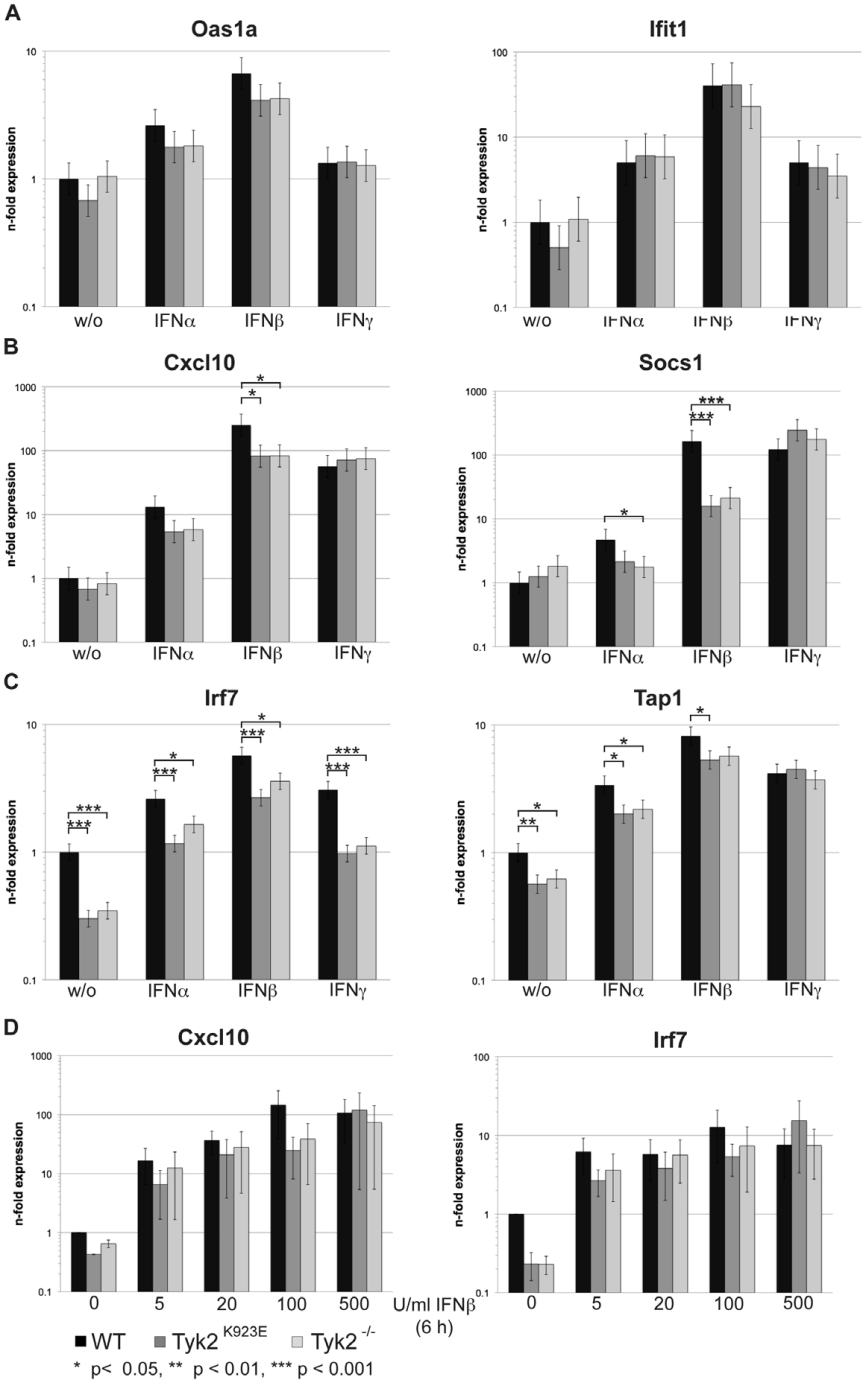
Transcriptional induction of IFN-responsive genes is similar in *Tyk2^K923E^* and *Tyk2^−/−^* cells. A.-C. WT, *Tyk2^−/−^* and *Tyk2^K923E^* BMMΦs were treated with IFNα (500 U/ml), IFNβ (100 U/ml) or IFNγ (100 U/ml) for 6 h or left untreated. Total RNA was extracted, reverse-transcribed and analysed by RT-qPCR for expression of *Oas1a*, *Ifit1* (A), *Cxcl1*, *Socs1* (B) and *Irf7, Tap1* (C). *Ube2D2* was used for normalization and expression levels were calculated relative to untreated WT cells. Data are derived from three independent experiments and depicted as mean values (+/− SE). D. WT, *Tyk2^−/−^* and *Tyk2^K923E^* BMMΦs were treated with indicated doses of IFNβ for 6 h. Target gene expression was determined as described in A-C. Mean values (+/− SD) derived from two independent experiments are depicted. Note that due to sample size a statistical analysis was not performed.

We previously reported that in macrophages many IFN response genes are less expressed at the basal, i.e. uninduced, state in the absence of TYK2. Upon inflammatory or viral stimulus the IFN response genes become less dependent on TYK2 [42], [43]. We analysed the expression of genes induced predominantly by IFN type I (*Ifit1*, *Oas1a*, *Ifi204*, *Irf7*) or by both IFN type I and II (*Tap1*, *Cxcl10*, *Socs1*, *Irgm1*). By means of RT-qPCR we monitored the levels of gene expression in WT, *Tyk2^−/−^* and *Tyk2^K923E^* BMMΦs either untreated or treated with IFNα, β or γ. In accordance with our previous observations, the response genes showed differential TYK2 dependency. We could divide the genes into three groups depending on their response to IFN treatment: (i) *Oas1a* and *Ifit1* were *Tyk2*-independent (Fig. 4A); (ii) *Cxcl10*, *Socs1* and *Irgm1* were *Tyk2*-dependent upon induction by IFN (Fig. 4B and data not shown); and (iii) *Tap1*, *Irf7* and *Ifi204* were *Tyk2*-dependent in the uninduced and induced states (Fig. 4C and data not shown). As anticipated from their similar STAT1-3 phosphorylation patterns, comparison of uninduced mRNA expression levels and IFNα/β inducibility showed no differences between *Tyk2^K923E^* and *Tyk2^−/−^* cells (Fig. 4A–C). Analysing the induction of *Cxcl10* and *Irf7* with IFNβ at different doses also revealed no differences between the mutant genotypes (Fig. 4D). Among the six genes analysed, only two (*Cxcl10* and *Socs1*, Fig. 4B) show significant dependence on kinase-active Tyk2 upon IFNβ (p<0.05), but not in response to IFNα. However a similar tendency (p<0.1) was seen for IFNα.

### *Tyk2^K923E^* and *Tyk2^−/−^* Mice Show Increased Susceptibility to Viral Infections

**Figure 5:**
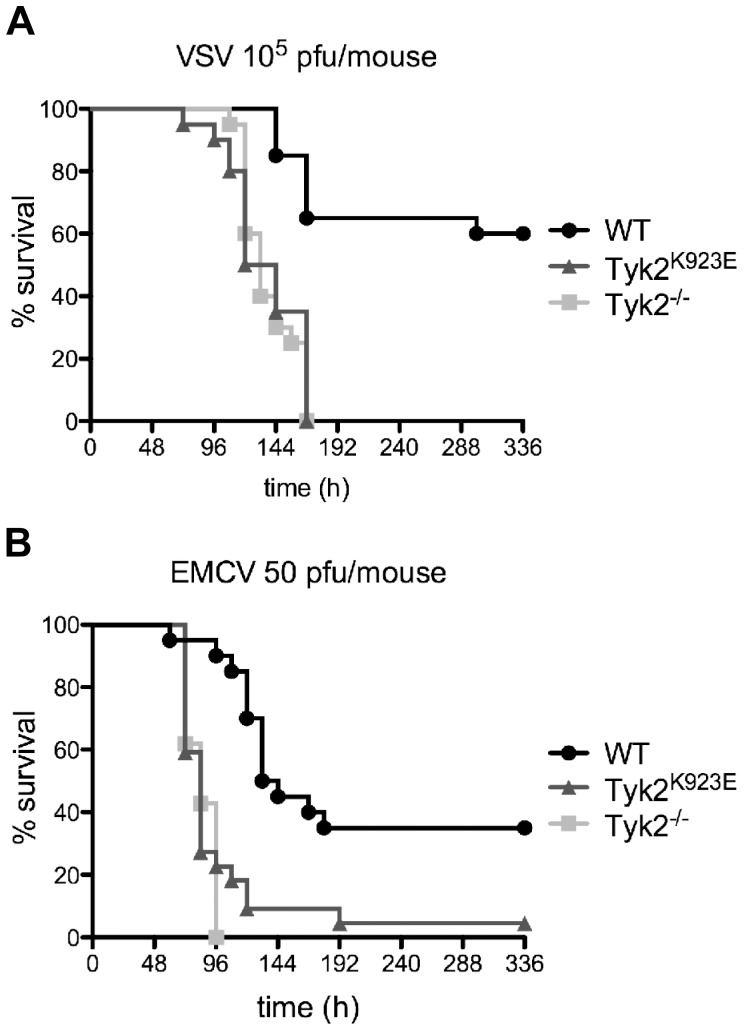
*Tyk2^−/−^* and *Tyk2^K923E^* mice show similarly increased susceptibility to virus infection. VSV (A) was administered intranasally (i.n.) and EMCV (B) intraperitoneally (i.p.). Mice were monitored twice daily for survival over a two-week period. Data are derived from two independent experiments (n = 20/genotype and n = 21/genotype for A and B, respectively).

To determine the immuno-competence of kinase-inactive *Tyk2* mice we infected mice with VSV and EMCV and monitored their survival. These viruses were chosen because they are predominantly cleared from the host through type I IFN-mediated mechanisms [44], [45]. We previously reported that upon i.v. administration WT and *Tyk2^−/−^* mice resist challenge by VSV [6], so we elected to use intranasal (i.n.) instillation, which is known to increase the susceptibility to lethal disease by 3-4-fold [46]. At a dose of 10^5^ pfu/mouse, >60% of the WT mice survived the challenge while none of the *Tyk2^−/−^* and *Tyk2^K923E^* mice survived for longer than d7 (Fig. 5A). *Tyk2*-mutant mouse strains were then infected i.p. with 50 pfu EMCV and survival was monitored. WT mice had a survival rate of 40%, whereas *Tyk2^−/−^* mice showed 100% mortality and *Tyk2^K923E^* mice showed a level of mortality that was slightly reduced, although the difference was not statistically significant (Fig. 5B). Thus kinase-active TYK2 is required for the antiviral responses against VSV and EMCV *in vivo*.

## Discussion

To dissect the enzymatic and putative non-enzymatic functions of TYK2 *in vivo* we generated knockin mice carrying *Tyk2* alleles (*Tyk2^K923E^*) with a point mutation that inactivates the ATP-binding pocket of the kinase domain (Fig. 1). Consistent with the findings from mice lacking the TYK2 protein [6], [7], [8], [9], loss of the kinase activity does not prevent mice from developing and reproducing normally. In an analogous approach, it was shown that JAK2-deficient and JAK2 kinase-inactive mice have the same phenotype, i.e. embryonal lethality [47].

*Tyk2* ^−/−^ and *Tyk2^K923E^* mice show no significant differences in activation of STAT1-4 induced by type I IFN, in transcriptional activation of IFN target genes or in survival upon viral infection (Figs. 3–5). This indicates that, with regard to type I IFN signalling, TYK2^K923E^ is not capable of complementing TYK2 deficiency nor does it act in an inhibitory manner. TYK2 kinase activity has been shown to be indispensable for IFNβ-induced apoptosis and for mitochondrial respiration in murine pro-B cells [29] and for IFNβ-induced gene expression and STAT3 activation in human fibroblasts [30], [48], [49]. The lack of biological activity in the type I IFN system is unlikely to result from limited availability of TYK2^K923E^ (see below) because a block of protein degradation and consequent increase of TYK2^K923E^ levels does not alter cells’ responsiveness to type I IFN (data not shown) and because inhibitory effects are only observed upon massive over-expression of TYK2^K923E^ in *Tyk2^−/−^* MEFs (C. Gausterer unpublished). In contrast, experiments with *Jak2* kinase-inactive mice revealed a mild dominant-negative phenotype of the mutated protein *in vivo*, while over-expression *in vitro* had an inhibitory effect [47].

Substitution of K923 into E leads to lower levels of TYK2 protein in various cells and organs. The drop is not caused by impaired transcriptional activity of the *Tyk2^K923E^* locus, as TYK2^K923E^ becomes more stable when the autophagosomal degradation pathway is blocked (Fig. 2). Lack of stability (or immunogenicity) in lymphocytes was previously observed in B10.Q/J mice carrying the TYK2^E775K^ mutation in the pseudokinase domain and this change was not reversed upon treatment with the proteasomal inhibitor MG-132 [9]. This finding supports our notion that mutated TYK2 is not exposed to proteasomal degradation. In contrast, *Jak2* kinase-inactive MEFs showed unperturbed stability of JAK2 [47]. Decreased TYK2 protein stability also seems to be a consequence of treating cells with JAK kinase inhibitors (see Fig. 2). We therefore propose including the assessment of JAK protein stability in future studies relating to the development or efficacy evaluation of JAK kinase inhibitors.

To date, JAKs and/or STATs have only been reported to be degraded by the proteasomal pathway under the control of SOCS (suppressors of cytokine signalling) [50], [51]. TYK2 is known to interact with SOCS1 [52], [53], [54], [55], although its proteasomal degradation *per se* has been studied only recently in the context of a viral IFN response evasion mechanism [56]. At the IFNAR, TYK2 is only destabilised when it is phosphorylated on binding to a ligand [54]. This suggests that SOCS-mediated proteasomal degradation may be specific for TYK2 activated by cytokines. Ligand-induced activation of TYK2 is governed by JAK1-mediated cross-phosphorylation of conserved tyrosine residues within the activation loop of the kinase domain. Additional tyrosines with putative regulatory functions that are potentially auto-phosphorylated have been identified by phosphoproteome mapping [5]. Although the lack of specific antibodies makes it impossible to test the idea, it is tempting to speculate that autophagosomal degradation of TYK2 is the cellular mechanism for regulating TYK2 levels under unstimulated physiological conditions, with auto-phosphorylation as one of the underlying regulatory mechanisms.

Interestingly, the basal TYK2 protein levels in WT cells differ considerably between organs and tissues (Fig. 2). TYK2 is widely reported to be ubiquitously expressed [2], [5]. The gene portal system BioGPS (http://biogps.org) lists some differences in *Tyk2* mRNA expression, with highest basal levels in lymphoid organs and cells. Nevertheless, reports of cell-specific variations in TYK2 amounts and their biological consequences are sparse. One paper correlated cell-type differences in available amounts of type I IFN signalling components, including TYK2, with intensity of response upon paracrine cytokine stimulation [57]. The authors suggested that cells are armed with elevated levels of signal transduction components to restrict the spread of pathogens. It remains to be established whether the disposable protein level is a molecular mechanism by which cells utilise TYK2 in different signalling cascades and organ-specific textures.

In conclusion, we report that kinase-inactive TYK2 cannot compensate for loss of TYK2 in type I IFN-mediated responses *in vitro* and *in vivo* and that inhibition of TYK2 kinase activity *in vivo* does not exacerbate the phenotype of loss of TYK2 with respect to virus susceptibility. Our future work will address the requirement for TYK2 kinase function in a tissue-restricted context and in cytokine response networks other than type I IFNs. To date, kinase-independent functions of TYK2 have only been described in human cells, in which catalytic activity is not required for IFNAR1 cell-surface anchoring and activation of PI3 kinase [25], [30], and in murine cells, in which kinase-inactive TYK2 is sufficient to enable basal mitochondrial respiration [29]. We show that inactivation of TYK2’s enzymatic activity by mutation or pharmaceutical intervention within the ATP binding pocket interferes with the protein’s stability. The translational research attempting to develop TYK2 kinase inhibitors [58], [59] should consider this potential side effect, which may be harnessed in future clinical applications. *Tyk2^K923E^* mice provide the first *in vivo* model for testing off-target effects of JAK inhibitors.

## Materials and Methods

### Ethics Statement

Mice were housed under specific pathogen-free conditions according to FELASA guidelines. All animal experiments were discussed and approved by the Ethics and Animal Welfare Committee of the University of Veterinary Medicine Vienna and conform the National Authority (Austrian Federal Ministry for Science and Research according to §8ff of Law of Animal Science and Experiments (Tierversuchsgesetz – TVG; refs. BMBWK-68.205/0240-BrGT/2005 and BMWF 68.205/0233-II/10b/2009).

### *Tyk2^K923E^* Gene Constructs

The expression plasmid pEFmTyk2 was cloned by inserting the murine *Tyk2* cDNA (NCBI GenBank: AF173032.1) into the polylinker of pEF-Zeo [60], controlling *Tyk2* expression by the elongation factor 1a promoter. For the single nucleotide exchanges in the *Tyk2* cDNA the PCR mutagenesis strategy described previously [19] was used. The nucleotide sequences of the primers were as follows (the mutated codons are underlined): mut-f 5′-GAGATGGTGGCCGTGGAGGCCCTTAAGGAAGGGTGCG-3′; mut-r 5′-CGCACCCTTCCTTAAGGGCCTCCACGGCCACCATCTC-3′; external-f 5′-AAGGGTTCCTAAAGAAGGGTTCATCAAATG-3′; external-r 5′-TCTGGATCCTGGAGCCCTG-3′. The resulting plasmid containing the mutated *Tyk2* cDNA was termed pEFmTyk2^K923E^.

To clone the gene-targeting vector the pKO-V920-Scrambler plasmid was used (Stratagene, La Jolla, CA; NCBI GeneBank: AF087567). The positive selection marker neomycin flanked by *loxP* sites was excised from the pKSloxPNT plasmid [61] (kindly provided by Alexandra L. Joyner of the New York University Medical Center, NY USA). The construct contained the long homologous arm with 8,039 bp (spanning exons 10-21, NCBI GeneBank: AC163637.4), the 2 kb neomycin cassette flanked by loxP sites and the short homologous arm with 1,254 bp (spanning exons 22 and 23) (Fig. 1B). Nucleotide exchanges into exon 20 of the murine *Tyk2* locus were introduced by site directed mutagenesis. A157820 was mutated to G157820 resulting in the amino acid exchange K>E in the ATP binding pocket of TYK2 (see above). An additional mutation from G21925 to T21925 was introduced, which did not affect the amino acid sequence but created an additional restriction site for the endonuclease BspTI.

### Purification of Recombinant GST-IFNAR*cyt* Fusion Protein

*E.coli* (XL1 blue) transformed with the pGEX-GSTIFNAR2*cyt* expression vector (kindly provided by Sandra Pellegrini, Institute Pasteur Paris, France) was grown in LB medium supplemented with 100 µg/ml ampicillin to an OD_600 nm_ of 0.6 to 0.8. Isopropyl β-D-1-thiogalactopyranoside (IPTG, 0.1 mM) was added and incubation continued for a further 1 hour with shaking at 30°C before cells were harvested by centrifugation. Bacterial pellets were resuspended in ice-cold PBS supplemented with 1% (v/v) Triton X-100, 1 mg/ml lysozyme and 1 mM PMSF. If not indicated otherwise all reagents were from ROTH (Karlsruhe Germany). The bacterial suspensions were frozen in liquid nitrogen and thawed on ice (3 cycles). Suspensions were sonicated four times for 30 seconds at 50% continuous power using the HD70 Sonopuls-ultrasonic-homogenizer (Bandelin Electronic GmbH & Co KG, Berlin Germany). Bacterial debris was pelleted (14000 g, 10 minutes at 4°C) and the supernatants used for affinity purification. A pre-packed column of glutathione sepharose 4B (GE Healthcare, Little Chalfont UK) was washed with 20 ml cold (4°C) PBS. The column’s gel bed was equilibrated with 6 ml PBS supplemented with 1% Triton X-100. Bacterial lysates were clarified by centrifugation and filtering (0.45 µm pore size) and applied to the column. The column was washed twice with 10 ml PBS, then bound material was eluted with 10 ml elution buffer (5 mM glutathione in 50 mM Tris-HCl pH 8.0). Fractions were collected and stored at –80°C until use for *in vitro* kinase assays. Purity was analysed by SDS-PAGE (10%) and visualization using GelCode Blue Stain Reagent (Pierce Biotechnology, Rockford IL USA) following the manufacturer’s instructions.

### *In vitro* Kinase Assay

U1A cells [4] were transiently transfected with expression vectors pEGFP (Clontech Laboratories, Inc., Palo Alto CA USA), pEFmTyk2 or pEFmTyk2^K923E^, applying the Superfect transfection technique (Qiagen, Hilden Germany). The *in vitro* kinase assay was performed as described previously [19], [22]. 18 hours post transfection cells were harvested and whole cell extracts prepared. 500 µg whole cell extracts were incubated with 4 µg anti-TYK2 antibody per sample with slow rotation at 4°C for 4 hours. 50 µl protein A slurry (50%; GE Healthcare, Little Chalfont UK) were added and incubated with slow rotation at 4°C for 2 hours. Immunoprecipitates were washed twice with 1 x lysis buffer and once with kinase buffer (50 mM NaCl, 5 mM MgCl_2_, 5 mM MnCl_2_, 0.2 mM NaVanadate, 10 mM HEPES pH 7.4). Pellets were resuspended in 50 µl kinase buffer supplemented with γ-^32^P-ATP (10 µCi per reaction; redivueTM adenosine 5‘-[γ-^32^P] triphosphate, triethylammonium salt; GE Healthcare, Little Chalfont UK). For assays of *in vitro* kinase activity on an exogenous substrate, the kinase reaction mixture was further supplemented with GST-IFNAR2*cyt* (1 µg/reaction). Kinase reactions were performed on a thermo-mixer (Eppendorf, Germany) shaking the tubes vigorously (1500 rpm) at 30°C for 5 minutes. Enzymatic activity was terminated by adding 75 µl 2×LSB. Samples were analysed by SDS-PAGE (7%) and autoradiography.

### Mice and Genotyping

C57BL/6N (WT) mice were purchased from Charles River Laboratories. *Tyk2^−/−^* (B6N.129P2-*Tyk2^tm1Biat^*) mice have been previously described [6] and were on C57BL/6N background. *Tyk2^K923E^* (B6;129P2-*Tyk2^tm3(K923E)Biat^* or B6N.129P2-*Tyk2^tm3(K923E)Biat^*) animals were on either mixed or C57BL/6N background. Data shown in Figs. 1–3 were from mixed background and data in Figs. 4 and 5 from pure bred mice. Experiments were performed with sex- and age-matched (8 to 12 week old) mice. Southern blot analysis was performed as described [32]. *Tyk2^K923E^* mice were screened by detection of the BspTI fragment using the primers 68.BspTIf 5′-CGAGATGGCTCAGCGGATAA-3′ and 89.K-E.rev 5′-TGGTCAGGCCAGGATAGTTC-3′ or by an assay designed to detect the loxP site (82.K-E.rev 5′-TGCACTGCGATTCCTAACAG-3′, 83.K-E.fwd 5′-CCAGGATCCAGAGACTCCAA-3′).

### Cell Culture

Bone marrow-derived macrophages (BMMΦs) were isolated and grown in the presence of CSF-1 derived from L929 cells as described previously [42]. Cells were cultivated in DMEM (PAA Laboratories, Pasching Austria) containing 10% heat-inactivated fetal bovine serum (FCS; Invitrogen Europe), 1 mM L-glutamine, 100 U/ml penicillin and 100 µg/ml streptomycin (Pen/Strep; Invitrogen Europe), 50 µM β-mercaptoethanol (β-ME; Invitrogen Europe) and 15% L929 cell-conditioned medium. Cells were cultivated for 8 days before the experiments. For isolation and culture of aCD3ε-activated T cells, spleens were removed, homogenized through a 100 µm cell strainer (BD Falcon™, BD Biosciences Europe, Erembodegem Belgium) and incubated with red cell lysis buffer (1 ml/spleen; Sigma Aldrich Austria) according to the manufacturer’s instructions. T cells were activated with aCD3ε-Ab (0.5 µg/µl; BD Pharmingen, BD Europe) and cultured for 3 days in RPMI 1640 (Invitrogen Europe) containing L-glutamine (PAA Laboratories, Pasching Austria) supplemented with 10% FCS, Pen/Strep (100 U/ml, 100 µg/ml), 50 µM β-ME, 1×non-essential amino acids (NAA; PAA Laboratories, Pasching Austria), 1 mM sodium pyruvate (Gibco, Invitrogen Europe) and 100 U/ml recombinant human IL-2 (rhIL-2, Proleukin, Novartis Austria). For NK/NKT cell culture, freshly isolated splenocytes were incubated with MACS DX5-coupled beads (Miltenyi Biotec, Bergisch Gladbach Germany) and subjected to positive selection using the appropriate column system. Subsequently, cells were cultured in RPMI 1640 containing L-glutamine supplemented with 10% FCS, Pen/Strep (100 U/ml, 100 µg/ml), 50 µM β-ME and 5000 U/ml rhIL-2 for 10 days.

### Cytokines and Inhibitors

Recombinant mouse IFNα (IFNαA  =  IFNα3), IFNβ and IFNγ were from Calbiochem® (Merck4Biosciences, Darmstadt Germany). Inhibitors: Z-Leu-Leu-Leu-aI (MG-132; Sigma Aldrich Austria), 3-methyladenine (3-MA; Sigma Aldrich Austria), bafilomycin A_1_ (Sigma Aldrich Austria) and JAK inhibitor I (panJak inhibitor; Calbiochem®, Merck4Biosciences, Darmstadt Germany).

### Isolation of Total RNA, Reverse Transcription and Quantitative PCR

Cells were lysed and organs homogenized in peqGOLD TriFast (PEQLAB, Erlangen Germany), RNA was isolated according to the manufacturer’s instructions. RNA purity was determined by spectrophotometry and agarose gel electrophoresis. 1 µg of RNA was reverse transcribed using the iSCRIPT cDNA synthesis kit (BIO-RAD Austria). Quantitative PCR was performed on an Eppendorf realplex4 or a Stratagene MX3000.

The primer and probes for *Ube2D2* have been described previously [42]. QuantiTect® Primer Assays (Qiagen, Hilden Germany) were reconstituted according to manufactureŕs instructions. The following assays were used: Mm_Socs_1_SG, Mm_Ifi204_2_SG, Mm_Tap1_1_SG, Mm_Oas1a_1_SG, Mm_Ifit1_1_2 and Mm_Irgm1_1_SG. Hot FIREPol® DNA Polymerase (Solis Biodyne, Tartu Estonia), EvaGreen® (Biotium Inc, Hayward CA USA) and dNTP Set (Fermentas ThermoScientific Austria) were used according to manufactureŕs instructions. The following additional primers were used: *Tyk2*-fwd: 5′-TGACAGGTGTCCCTGTGAGATCTAT-3′, *Tyk2-*rev: 5′-CTGGAGGATGGGCACAAGA-3′, *Tyk2* probe: 5′-TCCTTCCGGCCCACCTTCCAGA-3′ (FAM); *Irf7*-fwd: 5′-TCTTCCGAGAACTGGAGGAGTT-3′, *Irf7*-rev: 5′- TCTCCTTGGGCCTCCCTG-3′, *Irf7* probe: 5′-CTCGGAGGCGGCAAGGGTCA-3′ (FAM/BHQ1).

RT-qPCR data were analysed using realplex (Eppendorf, Vienna Austria) software and the standard curve method used for the calculation of relative expression levels as previously described [42], [43], [62]. Statistical analysis was undertaken with the software SPSS 17.0 (Mac OS-X). Data were log-transformed for approximate normality and analysed with a linear model (ANOVA) with genotype, treatment and interaction as factors. Appropriate contrasts were calculated (with SPSS) and resulting p-values are reported.

### Whole Cell Extracts (WCE), Immunoprecipitation (IP) and Western Blot Analysis (WB)

Cells were lysed in 50 mM Tris/HCl pH 8.0, 10% (v/v) glycerol, 25 mM EDTA, 150 mM NaCl (all from ROTH, Karlsruhe Germany), 2 mM DTT, 0.5% NP40 (Igepal CA-630), 25 mM sodium fluoride, 1 mM sodium vanadate, 0.5 mM PMSF, SIGMA*FAST* Protease Inhibitor (all from Sigma Aldrich Austria) and cell debris removed by centrifugation. For IPs, 1 or 3 mg protein/ml from freshly prepared whole cell extracts (WCE) were incubated with 2 µg/ml antibody at 4°C overnight. 50 µl Protein A Sepharose® CL-4B (50% v/v; GE Healthcare, Little Chalfont UK) was added and samples incubated with slow rotation at 4°C for 2 hours. Samples were washed three times with lysis buffer and resuspended in 50 µl 2×Laemmli sample buffer. Proteins were separated with SDS-PAGE and blotted onto nitrocellulose membranes (Hybond, GE Healthcare, Little Chalfont, UK). For IPs, the amount loaded per lane corresponds to 400 µg input cell lysate (derived from 1×10^6^–2×10^6^ cells). For WBs, 15 µg total cell lysate was loaded per lane (derived from 4×10^4^−8×10^4^ cells). PageRuler® Prestained Protein Ladder (Fermentas ThermoScientific Austria) was used as molecular weight standard. Membranes were probed with the indicated antibodies and the ECL Western blotting detection system (GE Healthcare, Little Chalfont, UK). Antibodies: anti-phospho-Stat1 (Tyr701), anti-Stat1, anti-phospho-Stat3 (Tyr705), anti-Stat3, anti-Stat4 (C46B10) and anti-phospho-tyrosine (Y1054/1055) Tyk2 (human) were from Cell Signaling Technology (New England Biolabs GmbH, Frankfurt Germany), anti-panERK (p42/p44 (ERK1/2) and 56 kDa and 85 kDa family members) and anti-phospho-Stat4 (Tyr693) from BD Transduction Laboratories (BD Biosciences Europe, Erembodegem Belgium), anti-NFκB (p65, CT), anti-phospho-Stat2 (Tyr689) and anti-Stat2 from Upstate® (Millipore, Billerica MA USA) anti-phospho-tyrosine (PY20), anti-heme oxygenase-1 (HO-1), anti-Jak1 (HR-785) and anti-Jak2 (C-20) from Santa Cruz Biotechnology® (Santa Cruz CA USA), anti-LC3 from Sigma Aldrich (Austria). TYK2 antibody (rabbit polyclonal) was raised against an N-terminal peptide of murine TYK2. Peroxidase-conjugated secondary antibodies (mouse and rabbit) were from GE Healthcare (Little Chalfont UK).

### Virus Infection

*Vesicular stomatitis virus* (VSV), Indiana strain was provided by T. Decker (MFPL, University of Vienna, Austria) and *Encephalomyocarditis virus* (EMCV) was from A. Pichlmair (CeMM, Austrian Academy of Sciences, Vienna Austria). Age- and sex-matched mice were infected intraperitonally (i.p.) with 50 plaque forming units (pfu) EMCV. For VSV challenges, age-matched female mice were anaesthetized by intraperitonal injection of ketamine-xylazine (100 mg ketamine/kg body weight and 4 mg xylazine/kg body weight; Ketasol and Xylasol, Graeub AG, Switzerland) and infected intranasally (i.n.) with 10^5^ pfu in 20 µl of phosphate-buffered saline (10 µl/nostril).
